# Factors Associated with Complementary Feeding Practices in Infants of Mothers with a History of High-Risk Pregnancy

**DOI:** 10.3390/nu18193183

**Published:** 2026-09-26

**Authors:** Reyna Sámano, Hugo Martínez-Rojano, Gabriela Chico-Barba, Estefania Aguirre-Minutti, María Eugenia Flores-Quijano, Carmen Flores-Cisneros, Jimena Hernández Rodríguez, Yosimara de la Cruz-Valle, Estela Godínez-Martínez, Jennifer Mier-Cabrera, Alexa Xiomara Toledo-Barrera

**Affiliations:** 1 Coordinación de Nutrición y Bioprogramación, Instituto Nacional de Perinatología, Secretaría de Salud, Mexico City 11000, Mexico; ssmr0119@yahoo.com.mx (R.S.); gabyc3@gmail.com (G.C.-B.); minuttiestefania@gmail.com (E.A.-M.); maru_fq@yahoo.com (M.E.F.-Q.); carmen.flores@inper.edu.mx (C.F.-C.); eygodinez@hotmail.com (E.G.-M.); jennifer.mier@gmail.com (J.M.-C.); alexa.xiomaratb@gmail.com (A.X.T.-B.); 2 Sección de Estudios de Posgrado e Investigación, Escuela Superior de Medicina del Instituto Politécnico Nacional, Mexico City 11340, Mexico; 3 Coordinación de Medicina Laboral, Instituto de Diagnóstico y Referencia Epidemiológicos (InDRE) “Dr. Manuel Martínez Báez”, Secretaría de Salud, Mexico City 01480, Mexico; 4 Laboratorio de Nutrición, Campus Lomas Verdes, Universidad del Valle de México, Paseo de las Aves 1, Naucalpan de Juárez 53220, Estado de Mexico, Mexico; jimenarzh@gmail.com; 5 Coordinación del Campus Regional del Taxco el Viejo, Zona Norte, Universidad Autónoma de Guerrero, Av. Javier Méndez Aponte 1 Fracc, Servidor Agrario, Chilpancingo de los Bravo 39070, Estado de Guerrero, Mexico; 20415559@uagro.mx; 6 Departamento De Salud, Universidad IberoamericanaProlongación Paseo de Reforma 880, Lomas de Santa Fe, Álvaro Obregón, Mexico City 01219, Mexico

**Keywords:** complementary feeding, breastfeeding, high-risk pregnancy, sociodemographic factors, Mexico, infant nutrition

## Abstract

*Background*: Evidence on complementary feeding (CF) practices among offspring of women with a history of high-risk pregnancy remains limited, despite their increased vulnerability to adverse maternal and neonatal outcomes. This study evaluated the associations between CF timing, first complementary foods, primary sources of information, and maternal, perinatal, and sociodemographic factors. *Methods:* A cross-sectional analysis was conducted involving 530 mother–child dyads with a history of high-risk pregnancy. Data regarding CF practices, maternal characteristics, neonatal outcomes, and sociodemographic factors were obtained from clinical records and structured interviews. Three outcomes were analyzed: timing of CF initiation, first food offered, and primary source of information. Bivariate analyses (Pearson’s chi-square) were followed by multivariable logistic regression adjusted for relevant covariates. *Results*: The introduction of CF occurred before 6 months of age in 22.3% of the cases (*n* = 118); 77.7% (*n* = 412) initiated it between 6 and 8 months of age. A breastfeeding duration of less than six months (aOR = 4.713, CI 95%; 2.829–7.838, *p* = 0.001) and a lack of financial support from parents or family (aOR = 1.830, CI 95%; 1.083–3.093, *p* = 0.024) demonstrated an independent association with early CF. Reliance on family members as the primary source of information (19.3%) correlated with shorter breastfeeding duration, suburban residence, and preterm birth. Vegetables were the most frequently introduced first food (69.6%), followed by fruit (28.1%); only 2.3% of infants received foods other than fruit and vegetables (cheese, yogurt, meat, or beans) during this initial stage. *Conclusions*: In this group of women with a history of high-risk pregnancies, the timing of complementary feeding initiation, the specific foods introduced, and the sources of information consulted were associated with maternal, perinatal, and sociodemographic factors. A breastfeeding duration of less than six months and a lack of financial support from the parents or family were identified as the main variables linked to the early introduction of complementary feeding. Furthermore, relying on the family as the primary source of information showed a close relationship with the timing of complementary feeding initiation.

## 1. Introduction

Promoting child health during the first 24 months of life depends crucially on two priority nutritional practices: breastfeeding and appropriate complementary feeding (CF), as established by the World Health Organization (WHO) and the United Nations Children’s Fund (UNICEF) [1].

CF is fundamental for ensuring optimal infant growth and development. It involves the introduction of solid or semi-solid foods that, alongside breast milk, ensure the infant’s nutrition [2]. From six months of age, exclusive breastfeeding is no longer sufficient; therefore, it is imperative to introduce safe, timely, and adequate foods to meet emerging metabolic requirements [1,3,4,5]. During this process, it is vital to avoid nutrient-poor foods and beverages, limit fruit juices, offer iron-rich foods (or fortified CF) daily, and implement responsive feeding practices [4].

Conversely, poor feeding practices—such as premature initiation, limited dietary diversity, or insufficient frequency—are closely linked to adverse nutritional outcomes, including underweight, wasting, and stunting [6]. Growth faltering during early childhood can trigger irreversible physical and cognitive damage, in addition to significantly increasing morbidity and mortality rates [7].

It has been documented across multiple countries that fewer than 25% of children in this age group achieve a Minimum Acceptable Diet (MAD) [4]. This issue is exacerbated in developing nations, where non-compliance with WHO guidelines is common [8], including low dietary diversity [9,10]. These suboptimal practices have even more significant consequences in infant groups with greater biological and nutritional vulnerability [11].

Among these groups, infants born to mothers with high-risk pregnancies constitute a particularly vulnerable population. Complications such as gestational diabetes, hypertensive disorders, obesity, and thyroid diseases are associated with a higher frequency of prematurity, fetal growth restriction, neonatal hospitalization, and breastfeeding difficulties. These conditions may alter the infant’s nutritional needs and influence feeding decisions during the first months of life [12].

While these infants typically require close clinical monitoring, it remains uncertain how maternal medical history influences CF decisions. The timing of initiation [13], the diversity and quality of the first foods offered [14], and the sources of information guiding these practices are of particular relevance in this population [15], as inadequate feeding could exacerbate nutritional deficiencies [14] or compromise growth during a critical developmental period [16].

However, although CF practices have been widely studied in the general population, there is limited evidence regarding the maternal, perinatal, and sociodemographic factors that determine these decisions in women with a history of high-risk pregnancy [17]. Understanding these determinants is essential for designing specific and effective nutritional interventions.

Therefore, the objective of this research was to evaluate the association between the timing of complementary feeding introduction, the initial foods offered, and the primary sources of information with maternal, perinatal, and sociodemographic factors in women with a history of high-risk pregnancy.

## 2. Materials and Methods

### 2.1. Study Design and Setting

A cross-sectional analysis nested within a prospective cohort of women with high-risk pregnancies was conducted, with a sample of 530 mother-infant dyads. Participants received prenatal care and delivered at the National Institute of Perinatology (INPer) in Mexico City, a tertiary care institution specializing in high-complexity obstetrics. Although the cohort was followed longitudinally from the third trimester through the postpartum period, the present study analyzed data from the first year of life to evaluate CF practices. Data collection was carried out between January 2021 and December 2024.

### 2.2. Participants

The study population consisted of women recruited during the third trimester of gestation and followed up until 24 months postpartum. A non-probabilistic consecutive sampling method was employed. The study included women with a history of high-risk pregnancy, defined by maternal, fetal, or obstetric conditions associated with increased morbidity or mortality [18]. These conditions included extreme maternal age (≤19 or ≥35 years), diabetes, pre-pregnancy obesity, renal diseases, autoimmune conditions, pulmonary or hepatic pathologies, severe anemia (Hemoglobin < 7.5 g/dL), Human Immunodeficiency Virus, thyroid disorders, and multiple pregnancy.

Inclusion criteria were: mothers with a live newborn delivered at more than 27 weeks of gestation, attendance of a minimum of three follow-up visits, and providing written informed consent. Gestational and maternal ages were verified through medical records. Sociodemographic information was collected during the third trimester; subsequently, at 6 and 12 months postpartum, questionnaires regarding breastfeeding and CF were administered.

### 2.3. Data Collection

A structured questionnaire was developed to collect information on infant feeding practices and associated factors. Prior to implementation, a pilot study was conducted with 30 women with similar characteristics (who were excluded from the final analysis) to assess the clarity and comprehension of the items. The instrument’s reliability yielded a Cronbach’s alpha coefficient of 0.83 and a concordance index (content validity) of 0.87.

Sociodemographic information, CF practices, and information sources were obtained through face-to-face interviews. Clinical data and obstetric events were extracted from medical records by trained personnel.

#### 2.3.1. Sociodemographic Information

Age was categorized as ≤19, 20–34.9, and ≥35 years. Educational level was classified as secondary school, high school, and university. Marital status was grouped into “married/cohabiting” and “single” (including widowed and divorced). Socioeconomic status was assessed using the Mexican Association of Market Intelligence Agencies (AMAI) index [19]. This classification system is validated for the Mexican population and continuously calibrated using official national statistics. Because self-reported income is frequently underreported, the AMAI index instead estimates socioeconomic level by measuring a household’s capacity to fulfill its core needs. Based on the responses, households were categorized into six socioeconomic tiers: A/B (highest), C+ (upper middle), C (average), D+ (below average), D (low), and E (lowest).

#### 2.3.2. Complementary Feeding Assessment and Breastfeeding

We assessed the infant’s age at the initiation of complementary feeding, the first food offered, and the primary source of maternal information. Based on WHO and UNICEF criteria [5], the initiation of complementary feeding was classified as timely (between 6 and 8 months of age, inclusive of infants starting exactly at 6 months), early (<6 months), and late (>8 months). Furthermore, breastfeeding from 0 to 6 months of age was assessed as ‘any breastfeeding’ (the provision of breast milk regardless of exclusivity). The continuation of breastfeeding was defined as maintaining breastfeeding beyond the first 6 months of life, alongside the introduction of complementary foods, up to two years of age or beyond [5].

#### 2.3.3. Food Insecurity

Household food insecurity (FI) was assessed using the Mexican Food Security Scale (Escala Mexicana de Seguridad Alimentaria, EMSA) [20], a validated instrument widely used in Mexico, including for the country’s official multidimensional poverty metrics by the National Council for the Evaluation of Social Development Policy (CONEVAL). The scale consists of 12 dichotomous (yes/no) questions directed at the mother. A score of 1 was assigned for each affirmative response and 0 for each negative response. Based on the total score, households were classified as experiencing FI if they scored >0 points, regardless of the presence of minors in the household.

### 2.4. Sample Size

An expected proportion of 50% for the timely introduction of CF was assumed, given the lack of prior data for this specific population. Based on a 95% confidence level, 4% precision, and a total population of 4000 annual births at INPer, the Epi Info™ 7 software determined a minimum sample size of 523 participants. The final sample consisted of 530 dyads.

### 2.5. Statistical Analysis

Data were analyzed using SPSS version 23 (IBM Corp., Armonk, NY, USA). The distribution of variables was assessed using the Kolmogorov–Smirnov test. Continuous variables were reported as mean ± standard deviation or median (interquartile range [IQR]), while categorical variables were presented as frequencies and percentages. Pearson’s chi-square test, Fisher’s exact test, Student’s *t*-test, or the Mann–Whitney U test were employed according to the nature and distribution of the data. Statistical significance was set at *p* < 0.05.

Three multivariable binary logistic regression models were constructed to evaluate: (1) early introduction of CF, (2) fruits as the first food, and (3) family as the primary source of information. Variables with *p* < 0.20 in the bivariate analysis, or those with proven clinical relevance, were included in the multivariable models. Results are expressed as adjusted odds ratios (aOR) with 95% confidence intervals (95% CI). Model fit was validated using the Hosmer–Lemeshow test and Nagelkerke’s *R*^2^.

For the multivariable analysis evaluating the type of first food introduced, the analytical sample was restricted to participants whose infants initiated complementary feeding with fruits or vegetables. Participants who reported another type of first food (*n* = 12) were excluded from this specific analysis, as they did not contribute to the outcome of interest. Models 1 and 3 included 530 participants, whereas Model 2 included 518.

### 2.6. Ethical Considerations

The study adhered to the Declaration of Helsinki and was approved by the Ethics Committee of the National Institute of Perinatology (protocol code 2020-1-15-INPer; date of approval: 20 December 2020). All participants provided written informed consent. In cases involving minors, consent was obtained from the legal guardian along with the ad-olescent’s assent. Privacy and anonymity were ensured through database coding.

## 3. Results

### 3.1. General Characteristics

Of the 530 mother-infant dyads included, the median maternal age was 29 years (P25: 23; P75: 35). Adolescent mothers accounted for 13% of the sample, 60% were between 20 and 34 years of age, and 27% were 35 years or older. During the first six months of life, 43.8% of the infants received breastfeeding, 19.4% received mixed feeding, and 36.8% were fed infant formula. The prevalence of breastfeeding decreased to 11.9% at 12 months and to 1.2% at 24 months. Regarding the newborns, 51.3% were female; additionally, 29.0% were born prematurely, 36.6% presented with low birth weight, and 24.1% had intrauterine growth restriction.

### 3.2. Introduction of Complementary Feeding

The introduction of complementary feeding (CF) occurred before 6 months of age in 22.3% of cases (*n* = 118) and between 6 and 8 months in the remaining 77.7% (*n* = 412). No child initiated CF after 8 months of age. Complementary feeding was initiated with cooked vegetables in 69.6% of the infants (*n* = 369) and with fresh or grated fruit (predominantly banana and apple) in 28.1%. The remaining 2.3% started with other food groups (cheese, yogurt, legumes, or meat). Overall, 61.7% of the newborns (*n* = 327) presented an adverse neonatal outcome, with a significant difference observed according to the source of guidance on CF (*p* = 0.035).

As shown in Table 1, the initiation of CF at or after 6 months was significantly more likely among mothers whose infants experienced an adverse neonatal outcome (*p* = 0.035), such as prematurity (*p* = 0.030), intrauterine growth restriction (*p* = 0.020), or hyperbilirubinemia (*p* = 0.006). This later initiation was also significantly associated with maternal pregestational obesity (*p* = 0.030). Similar trends were observed for mothers with pregestational hypothyroidism (*p* = 0.068) and those experiencing some degree of food insecurity (*p* = 0.055), although these associations did not reach statistical significance. Conversely, the introduction of CF before 6 months was significantly associated with relying on family members as the primary source of information (*p* < 0.001) and with the duration of breastfeeding < 6 months (*p* < 0.001). Detailed data on the remaining variables and their frequencies according to the timing of CF introduction are provided in Supplementary Table S1.

### 3.3. Initial Introduction of Solid Foods

Complementary feeding was initiated with cooked vegetables in 69.6% of the infants (*n* = 369) and with fresh or grated fruit (predominantly banana and apple) in 28.1%. The remaining 2.3% began with other food groups. As shown in Table 2, the initiation of complementary feeding with fruit was more frequent among mothers who lacked emotional support from a partner or family (*p* = 0.025) and those who relied on information from relatives (*p* = 0.056), although the latter trend did not reach statistical significance. Conversely, mothers with gestational diabetes were less likely to introduce fruit initially (*p* = 0.035). Further details on the remaining analyzed variables are provided in Supplementary Table S2.

### 3.4. Sources of Information

Of the 530 participating mothers, 75.1% (*n* = 398) received guidance on CF initiation from healthcare providers, while 24.9% (*n* = 132) were guided by a family member, most notably the grandmother. No significant differences were observed across several sociodemographic variables (socioeconomic status, occupation, maternal age, and parity), suggesting homogeneity between the groups in these aspects (Supplementary Table S3).

However, significant differences emerged regarding specific socioeconomic, clinical, and neonatal characteristics (Table 3). Mothers with lower educational attainment (*p* = 0.015), those relying on parents or family as their primary income source (*p* < 0.001), those who experienced anemia during pregnancy (*p* = 0.038), and those with preterm infants (*p* = 0.015) were more likely to identify family (primarily the maternal grandmother) as their main source of information. In contrast, for mothers whose infants had hyperbilirubinemia (*p* = 0.028), the primary source of information was medical personnel.

### 3.5. Factors Associated with the Initiation of Complementary Feeding

Table 4 presents the multivariable logistic regression models for the three evaluated outcomes:

In Model 1, a breastfeeding duration of less than six months was associated with almost five-fold higher odds of initiating CF before six months (aOR = 4.71). Similarly, lack of financial support from the parents or family (aOR = 1.83) and pre-pregnancy insulin resistance (aOR = 2.32) remained significantly associated with this outcome. In contrast, a history of hypothyroidism (aOR = 0.33), pre-pregnancy obesity (aOR = 0.38), intrauterine growth restriction (aOR = 0.53), and neonatal hyperbilirubinemia (aOR = 0.29) were associated with lower odds of early CF introduction.

In Model 2, mothers engaged in employment or academic activities had 1.55 times higher odds of initiating CF with fruits (aOR = 1.55). Likewise, lack of emotional partner or family support was associated with increased odds of offering fruits as the first food (aOR = 1.67).

Finally, in Model 3, breastfeeding for less than six months (aOR = 1.700), residing in suburban areas (aOR = 1.748), and preterm birth (aOR = 1.343) were associated with higher odds of the family being the primary source of information regarding complementary feeding. Conversely, neonatal hyperbilirubinemia was associated with lower odds for this outcome (aOR = 0.525).

## 4. Discussion

Three primary findings emerged from this cohort of women with a history of high-risk pregnancy. First, nearly one-quarter of mothers initiated CF before the recommended age. Second, a duration of breastfeeding <6 months and a lack of financial support from parents or family were the main factors associated with early initiation. Third, maternal and neonatal clinical conditions, coupled with sociocultural factors, were linked to both the sources of information and the selection of first foods. These results demonstrate that CF decisions in this population are related to a complex interplay between biological and social factors.

### 4.1. Timing of Complementary Feeding Initiation

In our study, 77.7% of the participants initiated complementary feeding between 6 and 8 months of age, in accordance with international recommendations, despite being a population with a history of high-risk pregnancies. However, this proportion is lower than those reported in countries such as Chile (92%), as well as in various regions of Europe [21,22,23,24,25] and Asia [26,27,28,29,30,31]. These findings suggest that substantial gaps in infant feeding practices persist, even among populations receiving specialized obstetric care. Furthermore, the early introduction of solid foods in nearly a quarter of the sample is clinically relevant due to its potential associations with breastfeeding duration and nutritional quality [32,33,34]. This pattern of introduction before six months is consistent with the disparities observed in low-resource settings in Mexico and Brazil [35,36].

Furthermore, 22.3% of the cohort demonstrated early initiation of CF, a practice linked to an elevated probability of obesity [37]. Although this proportion was low, it highlights a vulnerable subgroup that may warrant targeted interventions, as this early introduction is inversely correlated with the positive outcomes typically associated with breastfeeding [30,35,38,39,40].

### 4.2. Factors Associated with Early Initiation of CF

A duration of breastfeeding <6 months and a lack of financial support from parents or family were significantly associated with the early initiation of CF. Breastfeeding is correlated with a protective effect [15,41], a finding consistent with our study. Conversely, the lack of financial support from parents or family may reflect economic constraints or a premature return to work, both of which are linked to a mother’s reduced capacity to maintain breastfeeding [42]. These results partially differ from those reported by Wasihun et al., who linked early initiation to lower educational attainment [43], suggesting that the factors associated with CF vary according to the maternal-child healthcare system and the specific population context.

### 4.3. Association Between Clinical Conditions and Medical Follow-Up

Mothers with pregestational insulin resistance were found to be more likely to initiate CF early. This metabolic condition has been linked to alterations in lactogenesis II, which could correlate with lower milk production and the early introduction of foods to satisfy the infant [44,45,46,47]. However, since gestational diabetes did not maintain statistical significance after adjustment, this association should be considered a plausible but unproven hypothesis [48,49].

In contrast to previous studies [50,51,52,53], pregestational obesity, hypothyroidism, and a history of neonatal conditions, such as hyperbilirubinemia or intrauterine growth restriction (IUGR), demonstrated an inverse association with early initiation. A probable explanation is that these high-risk conditions are typically linked to closer and more frequent contact with healthcare services, a pattern that correlates with greater exposure to nutritional counseling [41,54,55,56]. Although the intensity of medical follow-up and nutritional counseling was not directly evaluated in this study, this finding highlights a potential connection between intensive clinical monitoring and opportunities to reinforce healthy feeding practices.

### 4.4. The Family as a Source of Information

Residing in suburban areas, preterm birth, and insufficient breastfeeding duration were associated with a greater reliance on the family as a source of information. This typically reflects lower exposure to professional counseling, prompting mothers to rely on the experience of close relatives, particularly mothers and grandmothers [4,57,58,59]. In the case of preterm infants, although they usually receive specialized follow-up, the family often assumes a predominant role in daily decisions once the infant is discharged. Therefore, educational interventions must be inclusive and target the entire infant care network [60,61].

### 4.5. Choice of First Food Offered

Although guidelines prioritize the early introduction of iron-rich foods [62,63], the introduction of vegetables (69.6%) and fruits (28.1%) predominated in our cohort. Only 2.3% of infants initiated complementary feeding with foods other than fruits or vegetables. This discrepancy suggests that fruits and vegetables continue to be culturally perceived as the only ‘safe’ starter foods [64]. While this pattern limits the initial exposure to iron-rich sources, further longitudinal studies are needed to determine if it is associated with a long-term delay in the intake of critical micronutrients, as subsequent dietary intake and iron status were not assessed in this study [65]. The tendency of mothers with gestational diabetes to introduce vegetables could be linked to greater nutritional awareness acquired during the management of their condition, although this association requires confirmation through longitudinal studies [66].

### 4.6. Strengths and Limitations

This study presents several limitations. First, its cross-sectional design precludes the establishment of causal relationships, and its single-center nature restricts the external validity of the findings. Furthermore, data collection from electronic medical records may introduce bias or misclassification. However, as its primary strength, this work stands out as one of the first in Latin America to simultaneously assess the timing of complementary feeding introduction, the types of food introduced, and the sources of information within a high obstetric risk population, integrating both biological and sociodemographic associated factors.

### 4.7. Future Perspectives

Moving forward, research on complementary feeding must center on precision nutrition and its potential link to early-life chronic disease prevention. It is essential to mitigate conditions like allergies and childhood obesity—often driven by the premature introduction of juices and ultra-processed, high-sugar purées—while always prioritizing the infant’s pace of maturation and neurodevelopment.

## 5. Conclusions

In this cohort of women with a history of high-risk pregnancies, the initiation of complementary feeding (CF), the specific foods introduced, and the sources of information consulted were associated with maternal, perinatal, and sociodemographic factors. A breastfeeding duration of less than six months and a lack of financial support from the parents or family were identified as the main factors associated with the early introduction of CF. Furthermore, the family played a crucial role as a source of information, showing a close relationship with the timing of CF initiation.

Although most mothers initiated CF at the recommended age, the limited inclusion of iron-rich foods at this initial stage reveals a critical deficiency in infant feeding practices. These findings underscore the need for comprehensive nutritional counseling interventions—directed at both mothers and their family support networks—to optimize feeding practices during maternal and child follow-up in high-risk populations.

Despite the considerable size of the cohort, the results are limited to a population of mothers with high-risk pregnancies, precluding their extrapolation to the general population.

## Figures and Tables

**Table 1:** Neonatal, maternal, and sociodemographic characteristics according to the timing of complementary feeding initiation (≥6 months vs. <6 months of age), *n* = 530.

Variable	≥6 Months (*n* = 412) *	<6 Months (*n* = 118)	*p*-Value
Sociodemographic Factors			
Food insecurity			0.055
Food security, *n* = 473	362 (88)	111 (94)	
Food insecurity, *n* = 57	50 (12)	7 (6)	
Source of CF guidance			0.001
Healthcare professionals, *n* = 398	333 (81)	65 (55)	
Family as a source of information, *n* = 132	79 (19)	53 (45)	
Breastfeeding duration			0.001
<6 months, *n* = 298	204 (49)	94 (80)	
≥6 months, *n* = 232	208 (51)	24 (20)	
Neonatal complications			0.035
None, *n* = 203	148 (36)	55 (47)	
≥1 complication, *n* = 327	264 (64)	63 (53)	
Preterm birth			0.030
Yes, *n* = 179	149 (36)	30 (25)	
No, *n* = 351	263 (64)	88 (75)	
Intrauterine growth restriction			0.020
Yes, *n* = 128	109 (26)	19 (16)	
No, *n* = 402	303 (74)	99 (84)	
Neonatal hyperbilirubinemia			0.006
Yes, *n* = 89	79 (19)	10 (9)	
No, *n* = 441	333 (81)	108 (91)	
Pregestational maternal risk factors			
Pregestational obesity			0.030
Yes, *n* = 125	106 (26)	19 (16)	
No, *n* = 405	306 (74)	99 (84)	
Gestational weight gain			0.040
Adequate/insufficient, *n* = 330	247 (60)	83 (70)	
Excessive, *n* = 200	165 (40)	35 (30)	
Pregestational hypothyroidism			0.068
Yes, *n* = 61	53 (13)	8 (7)	
No, *n* = 469	359 (87)	110 (93)	

*p*-values were determined using Pearson’s chi-square test; CF: Complementary Feeding; Data are presented as *n* (%). * For the statistical analysis, the early initiation of complementary feeding was defined as the introduction of solid foods before 6 months of age, and adequate initiation as occurring between 6 and 8 months. The late initiation category was not evaluated, as no foods were introduced after 8 months of age in any case.

**Table 2:** Comparison of maternal and sociodemographic characteristics according to the first complementary food offered (vegetables vs. fruits), * *n* = 518.

Variables	Vegetables, *n* = 369	Fruits, *n* = 149	*p*-Value
Sociodemographic factors			
Source of guidance on CF			0.056
Family as a source of information, *n* = 127	82 (22)	45 (30)	
Health personnel, *n* = 391	287 (78)	104 (70)	
Emotional support from a partner or family			0.025
Yes, *n* = 273	206 (56)	67 (45)	
No, *n* = 245	163 (44)	82 (55)	
Maternal factors			
Pregestational obesity			0.073
Yes, *n* = 121	94 (25)	27 (18)	
No, *n* = 397	275 (75)	122 (82)	
Gestational diabetes			0.035
Yes, *n* = 67	55 (15)	12 (8)	
No, *n* = 451	314 (85)	137 (92)	

*p*-values determined by Pearson’s chi-square test; CF: Complementary Food; * The analysis was conducted on 518 of the 530 children in the cohort; 12 participants were excluded for initiating complementary feeding with foods other than fruits or vegetables; data are presented as *n* (%).

**Table 3:** Maternal and infant characteristics according to the source of information on complementary feeding (family vs. healthcare providers), *n* = 530.

Variables	Family, *n* = 132	Healthcare Providers, *n* = 398	*p*-Value
Sociodemographic factors			
Educational level			0.015
Secondary or higher, *n* = 488	115 (87)	373 (94)	
Primary education or lower, *n* = 42	17 (13)	25 (6)	
Primary income source			0.001
Self or spouse, *n* = 416	81 (61)	335 (84)	
Parents or family, *n* = 114	51 (39)	63 (16)	
Duration of breastfeeding			0.039
<6 months, *n* = 298	64 (48)	234 (59)	
≥6 months, *n* = 232	68 (52)	164 (41)	
CF initiation			<0.001
<6 months, *n* = 118	53 (40)	65 (16)	
≥6 months, *n* = 412	79 (60)	333 (84)	
Neonatal outcomes			
Preterm birth			0.015
Yes, *n* = 179	56 (43)	123 (31)	
No, *n* = 351	76 (57)	275 (69)	
Neonatal hyperbilirubinemia			0.028
Yes, *n* = 89	14 (11)	75 (19)	
No, *n* = 441	118 (89)	323 (81)	
Maternal outcomes			
Gestational diabetes			0.075
Yes, *n* = 68	11 (8)	57 (14)	
No, *n* = 462	121 (92)	341 (86)	
Anemia			0.038
Yes, *n* = 100	33 (25)	67 (17)	
No, *n* = 430	99 (75)	331 (83)	

*p*-value obtained using Pearson’s chi-square test; CF: Complementary Food; data are expressed as *n* (%).

**Table 4:** Multivariable logistic regression models for factors associated with the timing of introduction, first food, and source of information on complementary feeding *.

Variable	aOR	95% IC	*p*-Value
Model 1. Factors associated with early introduction of CF (<6 months), *n* = 530			
Food insecurity	0.464	0.191–1.124	0.089
Duration of breastfeeding <6 months	4.713	2.829–7.838	0.001
Lack of financial support from parents or family	1.830	1.083–3.093	0.024
Pregestational insulin resistance	2.325	1.072–5.045	0.033
Pregestational hypothyroidism	0.333	0.145–0.763	0.009
Pregestational obesity	0.389	0.212–0.714	0.002
Gestational diabetes	1.494	0.776–2.875	0.229
Intrauterine growth restriction	0.538	0.302–0.958	0.035
Neonatal hyperbilirubinemia	0.294	0.140–0.618	0.001
Model 1: The multivariable logistic regression model was statistically significant (χ^2^ = 79.861; *df* = 9; *p* < 0.001), with a Nagelkerke *R*^2^ of 0.214. Furthermore, the Hosmer–Lemeshow test indicated a good model fit (χ^2^ = 3.937; *df* = 7; *p* = 0.787).			
Model 2. Factors associated with offering fruit vs. vegetables at the start of complementary feeding, *n* = 518			
Occupation: studying or working	1.554	1.006–2.401	0.047
Primary education level or lower	1.722	0.871–3.405	0.118
Lack of emotional partner or family support	1.673	1.129–2.478	0.010
Gestational diabetes	0.528	0.270–1.033	0.062
Pregestational obesity	0.663	0.406–1.085	0.102
Inadequate GWG	1.414	0.943–2.150	0.105
Model 2: The multivariable logistic regression model was statistically significant (χ^2^ = 20.786; *df* = 6; *p* = 0.002) and showed an adequate goodness of fit according to the Hosmer–Lemeshow test (χ^2^ = 5.804; *df* = 8; *p* = 0.669), with a Nagelkerke *R*^2^ of 0.056.			
Model 3. Factors associated with the family as a source of information on complementary feeding, *n* = 530			
Duration of breastfeeding <6 months	1.700	1.120–2.581	0.027
Primary education level or lower	1.761	0.880–3.525	0.110
Residence in suburban areas	1.748	1.125–2.715	0.024
Preterm birth	1.343	1.007–1.792	0.045
Gestational diabetes	2.697	0.889–3.240	0.101
Neonatal hyperbilirubinemia	0.525	0.280–0.983	0.044
Anemia	1.622	0.994–2.648	0.053
Model 3: The multivariable logistic regression model was statistically significant (χ^2^ = 25.783; *df* = 7; *p* = 0.001), with a Nagelkerke *R*^2^ of 0.070. The Hosmer–Lemeshow test indicated an adequate model fit (χ^2^ = 11.221; *df* = 8; *p* = 0.190).			

* For the statistical analysis, the early initiation of complementary feeding was defined as the introduction of solid foods before 6 months of age, and adequate initiation as occurring between 6 and 8 months. The late initiation category was not evaluated, as no foods were introduced after 8 months of age in any case. Participants who reported another type of first food (*n* = 12) were excluded from this specific analysis, as they did not contribute to the outcome of interest. Models 1 and 3 included 530 participants, whereas Model 2 included 518. aOR: adjusted odds ratio; 95% CI: 95% confidence interval; GWG: gestational weight gain. Each model represents an independent multivariable binary logistic regression. Candidate variables were selected based on bivariate analysis (*p* < 0.20) and their clinical relevance as potential confounders.

## Data Availability

The data presented in this study are available upon reasonable request from the corresponding author.

## Supplementary Materials

The following supporting information can be downloaded at: https://www.mdpi.com/article/10.3390/nu18193183/s1, Table S1: Neonatal, maternal, and sociodemographic characteristics according to the timing of complementary feeding initiation (≥6 months vs. <6 months of age); Table S2: Comparison of maternal and sociodemographic characteristics according to the first complementary food offered (vegetables vs. fruits), * *n* = 518; Table S3: Maternal and infant characteristics according to the source of information on complementary feeding (family vs. healthcare providers), *n* = 530.

## Abbreviations

AMAI	Mexican Association of Market Intelligence Agencies
CF	Complementary Feeding
EBF	Exclusive breastfeeding
GWG	Gestational Weight Gain
INPer	National Institute of Perinatology
IUGR	Intrauterine Growth Restriction
UNICEF	United Nations Children’s Fund
WHO	World Health Organization
