# Supplementary material for: Factors Associated with Complementary Feeding Practices in Infants of Mothers with a History of High-Risk Pregnancy
Source: Nutrients. 2026 Sep 26;18(19):3183. doi: 10.3390/nu18193183 (PMC13666572; doi:10.3390/nu18193183)
Supplement: Supplementary file 1 [file nutrients-18-03183-s001.zip › nutrients-4533961-supplementary.pdf]

Supplementary tables

Table S1. Neonatal, maternal, and sociodemographic characteristics according to the timing of complementary feeding initiation (≥ 6 months vs. < 6 months of age)

**Variables**	**≥ 6 months, *n* = 412**	**< 6 months, *n* = 118**	***p*-value**
**Food Insecurity**			0.055
Absent, *n*=473	362 (88)	111 (94)	
Present, *n*= 57	50 (12)	7 (6)	
**Duration of breastfeeding**			0.001
< 6 months (a), *n*=298	204 (49)	94 (80)	
≥ 6 months, *n* = 232	208 (51)	24(20)	
**Source of CF Guidance (b)**			0.001
Family member (mother), n= 132	79 (19)	53 (45)	
Healthcare professionals, n = 398	333 (81)	65 (55)	
**First Complementary Food***			0.725
Vegetables, n=369	290 (72)	79 (70)	
Fruits, n=149	115 (28)	34 (30)	
**Occupation**			0.863
Homemaker, *n*= 392	304 (74)	88 (75)	
Student/Employed, *n*=138	108 (26)	30 (25)	
**Primary income source**			0.092
Self or Spouse, *n* = 416	330 (80)	86 (73)	
Parents or family (c), *n* =114	82 (20)	32 (27)	
**Emotional support from a partner or family**			0.815
Yes, n=279	218 (53)	61 (52)	
No, n=251	194 (47)	57 (48)	
**Socioeconomic Status**			0.719
Lower-middle, n=352	272 (66)	80 (68)	
Very Low, n=178	140 (34)	38 (32)	
**Educational Level**			0.072
Secondary or higher, n=488	384 (93)	104 (88)	
Primary or less, n=42	28 (7)	14 (12)	
**Marital status**			0.868
Married, n = 365	82 (69)	283 (69)	
Single, n = 165	36 (31)	129 (31)	
**Age (years)**			0.688
< 19 n=68	55 (13)	13 (11)	
20-34, n=318	248 (60)	70 (59)	
≥ 35, n=144	109 (27)	35 (30)	
**Residence**			0.724
Mexico City (urban), n=295	231 (56)	64 (55)	
Suburban areas, n=235	181 (44)	54 (45)	
**Parity**			0.574
Multigravida, n=164	125 (30)	39 (33)	
Primigravida, n=366	287 (70)	79 (67)	
**Pre-pregnancy Risk**			0.575

Without conditions, n=244	187 (45)	57 (48)	
With conditions, n=286	225 (55)	61 (52)	
**Gestational Complications**			0.332
Without complications, n=149	120 (29)	29 (25)	
With complications, n=381	292 (71)	89 (75)	
**Mode of Delivery**			0.953
Vaginal delivery, n=259	202 (49)	57 (48)	
Cesarean section, n=271	210 (51)	61 (52)	
**Gestational weight gain**			0.040
Adequate/insufficient, *n* = 330	247 (60)	83 (70)	
Excessive, *n* = 200	165 (40)	35 (30)	
**Insufficient Weight Gain**			0.383
Sufficient, n=369	283 (67)	86 (73)	
Insufficient, n=161	129 (31)	32 (27)	
**Neonatal Complications**			0.035
None, n=203	148 (36)	55 (47)	
≥ 1 complication, n=327	264 (64)	63 (54)	
**Preterm Birth**			0.030
No, n=351	263 (64)	88 (75)	
Yes, n=179	149 (36)	30 (25)	
**Macrosomia**			0.479
Absent, n=501	391 (95)	110 (93)	
Present, n= 29	21 (5)	8 (7)	
**IUGR (d)**			0.020
Absent, n=402	303 (74)	99 (84)	
Present; n=128	109 (26)	19 (16)	
**Low Birth Weight**			0.119
Absent, n=336	254 (62)	82 (70)	
Present, n=194	158 (38)	36 (30)	
**Neonatal hyperbilirubinemia**			0.006
Absent, n=441	333 (81)	108 (91)	
Present, n=89	79 (19)	10 (9)	
**Congenital Defects**			0.663
Absent, n= 460	359 (87)	101 (86)	
Present, n=70	53 (13)	17 (14)	
**Pregestational obesity**			0.030
Absent, n=405	306 (74)	99 (84)	
Present, n=125	106 (26)	19 (16)	
**Insulin resistance**			0.091
Absent, n=487	383 (93)	104 (88)	
Present, n=43	29 (7)	14 (12)	
**Gestational Diabetes**			0.372
Absent, n=462	362 (88)	100 (85)	
Present, n=68	50 (12)	18 (15)	
**Hypothyroidism**			0.068
Absent, *n* = 469	359 (87)	110 (93)	
Present, *n* = 61	53 (13)	8 (7)	
**Pre-/Eclampsia, PIH (e)**			0.726

Absent, *n* = 416	322 (78)	94 (80)	
Present, *n* = 114	90 (22)	24 (20)	
**Anemia**			0.643
Absent, *n* = 430	336 (82)	94 (80)	
Present, *n* = 100	76 (18)	24 (20)	

Note: (a) Refers to breastfeeding duration; (b) CF: Complementary Feeding; (c) Primary financial provider; (d) IUGR: Intrauterine Growth Restriction; (e) PIH: Pregnancy-Induced Hypertension; * The analysis was conducted on 518 of the 530 children in the cohort; 12 participants were excluded for initiating complementary feeding with foods other than fruits or vegetables; Data are presented as n (%). *p*-value by chi square of Pearson.

Table S2. Comparison of maternal and sociodemographic characteristics according to the first complementary food offered (vegetables vs. fruits), *n = 518

Variables	Vegetables, *n* = 369	Fruits, *n* = 149	*p*-value
**Duration of breastfeeding**			0.884
≤ 5 months, *n* = 292	207 (56)	85 (57)	
≥ 6 months, *n* = 226	162 (44)	64 (43)	
**Food Insecurity**			0.229
Absent, n=463	326 (88)	137 (92)	
Present, n=55	43 (12)	12 (8)	
**Source of Guidance on CF (a)**			0.056
Health personnel, *n* = 391	287(78)	104 (70)	
Family member, *n* = 127	82 (22)	45 (30)	
**Occupation**			0.113
Homemaker, *n* = 383	280 (76)	103 (69)	
Student/Employed, *n* = 135	89 (24)	46 (31)	
**Socioeconomic status**			0.383
Lower-middle, n=345	250 (68)	95 (64)	
Very low, n=173	119 (32)	54 (36)	
**Educational Level**			0.130
Secondary or higher, *n* = 477	344 (93)	133 (89)	
Primary or less, *n* = 41	25 (7)	16 (11)	
**Marital status**			0.948
Married, n = 357	254 (69)	103 (69)	
Single, n = 161	115 (31)	46 (31)	
**Age (years)**			0.627
< 19, n=65	47 (13)	18 (12)	
20-34, n=314	219 (59)	95 (64)	
≥ 35, n=139	103 (28)	36 (24)	
**Emotional support from a partner or family**			0.025
Yes, *n* = 273	206 (56)	67 (45)	
No, *n* = 245	163 (44)	82 (55)	
**Primary income source**			0.932
Self or Spouse, *n* = 408	291 (79)	117 (78)	
Parents or Family, *n* = 110	78 (21)	32 (22)	
**Residence**			0.821
Mexico City (urban), *n* =288	204 (55)	84 (56)	
Suburban areas, *n* =230	165 (45)	65 (44)	

**Parity**			0.265
Multigravida, *n* = 161	120 (33)	41 (27)	
Primigravida, *n* = 357	249 (67)	108 (73)	
**Pre-pregnancy Risk**			0.692
Without conditions, *n* = 240	173 (47)	67 (45)	
With conditions, *n* = 278	196 (53)	82 (55)	
**Gestational Risk**			0.501
Without complications, *n* = 149	103 (28)	46 (31)	
With complications, *n* = 369	266 (72)	103 (69)	
**Mode of delivery**			0.111
Vaginal delivery, *n* = 254	189 (51)	65 (44)	
Cesarean section, *n* = 264	180 (49)	84 (66)	
**Excessive Weight Gain**			0.234
Adequate/Low weight, n = 320	222 (60)	98 (66)	
Excessive, n = 198	147 (40)	51 (34)	
**Insufficient Weight Gain**			0.195
Sufficient, n=362	264 (71)	98 (66)	
Insufficient, n=156	105 (29)	51 (34)	
**Neonatal Complications**			0.726
None, n = 199	140 (38)	59 (40)	
≥ 1 complication, n = 319	229 (62)	90 (60)	
**Preterm Birth**			0.712
Absent, n = 371	266 (72)	105 (70)	
Present, n = 147	103 (28)	44 (30	
**Macrosomia**			
Absent, n = 489	347 (94)	142 (95)	0.571
Present, n = 29	22 (6)	7 (5)	
**IUGR (b)**			
Absent, n = 393	286 (78)	107 (72)	0.170
Present, n = 125	83 (22)	42 (28)	
**Low Birth Weight**			0.479
Absent, n = 332	240 (65)	92 (62)	
Present, n = 186	129 (35)	57(38)	
**Jaundice**			0.265
Absent, n = 430	302 (82)	128 (86)	
Present, n = 88	67 (18)	21 (14)	
**Congenital Defects**			0.202
Absent, n = 450	325 (88)	125 (84)	
Present, n = 68	44 (12)	24 (16)	
**Maternal factors**			
**Obesity**			0.073
Absent, n = 397	275 (75)	122 (82)	
Present, n = 121	94 (25)	27 (18)	
**Insulin resistance**			0.744
Absent, n = 476	340 (92)	136 (91)	
Present, n = 42	29 (8)	13 (9)	
**Gestational Diabetes**			0.035
Absent, n = 451	314 (85)	137 (92)	

Present, n = 67	55 (15)	12 (8)	
**Hypothyroidism**			0.662
Absent, n = 457	327 (89)	130 (87)	
Present, n = 61	42 (11)	19 (13)	
**Pre-/Eclampsia, PIH (c)**			0.575
Absent, n = 409	289 (78)	120 (80)	
Present, n = 109	80 (22)	29 (20)	
**Anemia**			0.560
Absent, n = 423	299 (81)	124 (83)	
Present, n = 95	70 (19)	25 (17)	

(a) CF: Complementary Feeding; (b) IUGR: Intrauterine Growth Restriction; (c) PIH: Pregnancy-Induced Hypertension; *p*-values determined by Pearson's chi-square test; * The analysis was conducted on 518 of the 530 children in the cohort; 12 participants were excluded for initiating complementary feeding with foods other than fruits or vegetables; Data are presented as n (%).

Table S3. Maternal and infant characteristics according to the source of information on complementary feeding (family vs. healthcare providers), *n* = 530

**Variables**	**Family members, *n* = 132**	**Healthcare workers, *n* = 398**	***p*-value**
**Food Insecurity**			0.559
Absent, n=473	116 (88)	357 (90)	
Present, n= 57	16 (12)	41 (10)	
**CF Initiation (a)**			<0.001
< 6 months, n = 118	53 (40)	65 (16)	
≥ 6 months, n = 412	79 (60)	333 (84)	
**Duration of breastfeeding**			0.039
< 6 months, *n* = 298	64 (48)	234 (59)	
≥ 6 months, *n* = 232	68 (52)	164 (41)	
**Occupation**			0.588
Homemaker, n=392	100 (76)	292 (73)	
Student/Employed, n=138	32 (24)	106 (27)	
**Socioeconomic Status**			0.321
Lower-middle, n=352	83 (63)	269 (68)	
Very low, n=178	49 (37)	129 (32)	
**Educational Level**			0.015
Secondary or higher, *n* = 488	115 (87)	373 (94)	
Primary or less, *n* = 42	17 (13)	25 (6)	
**Marital status**			0.086
Married, n = 365	83 (63)	282 (71)	
Single, n = 165	49 (37)	116 (29)	
**Age**			0.799
< 19 years, n=68	18 (14)	50 (13)	
20 34 years, n=318	81 (61)	237 (59)	
≥ 35 years, n=144	33 (25)	111 (29)	
**Residence**			0.054
Mexico City (urban), *n* = 295	83 (63)	212 (53)	

Suburban areas, *n* = 235	49 (37)	186 (47)	
**Emotional support from a partner or family**			0.367
Yes, n=279	65 (49)	214 (54)	
No, n=251	67 (51)	184 (46)	
**Primary income source**			0.001
Self or spouse, n=416	81 (61)	335 (84)	
Parents or Family, n=114	51 (39)	63 (16)	
**Parity**			0.403
Multigravida, n=164	37 (28)	127 (32)	
Primigravida, n=366	95 (72)	271 (68)	
**Pre-pregnancy Risk**			0.394
Without conditions, n=244	65 (49)	179 (45)	
With conditions, n=286	67 (51)	219 (55)	
**Gestational Risk**			0.359
Without complications, n=149	33 (25)	116 (29)	
With complications, n=381	99 (75)	282 (71)	
**Mode of Delivery**			0.862
Vaginal delivery, *n*=259	65 (49)	194 (49)	
Cesarean section, *n*=271	67 (51)	204 (51)	
**Excessive Weight Gain**			0.319
Adequate/Low weight, *n*=330	87 (66)	243 (61)	
Excessive, *n*=200	45 (34)	155 (39)	
**Insufficient Weight Gain**			0.266
Sufficient, *n*=369	97 (74)	272 (68)	
Insufficient, *n*=161	35 (26)	126 (32)	
Newborn Complications			0.477
None, n=203	54 (41)	149 (37)	
≥ 1 complication, n=327	78 (59)	249 (63)	
**Preterm Birth**			0.015
Absent, n=351	76 (57)	275 (69)	
Present, n=179	56 (43)	123 (31)	
**Macrosomia**			0.731
Absent, n=501	124 (94)	377 (95)	
Present, n=29	8 (6)	21 (5)	
**IUGR (b)**			0.619
Absent, n = 402	98 (74)	304 (76)	
Present, n = 128	34 (26)	94 (24)	
**Low Birth Weight**			0.576
Absent, n=336	81 (61)	255 (64)	
Present, n=194	51 (39)	143 (36)	
**Neonatal hyperbilirubinemia**			0.028
Absent, n=441	118 (89)	323 (81)	
Present, n=89	14 (11)	75 (19)	
**Congenital Defects**			0.671
Absent, n=460	116 (88)	344 (86)	
Present, n=70	16 (12)	54 (14)	
**Pregestational obesity**			0.975
Absent, n=405	101 (77)	304 (76)	

Present, n=125	31 (23)	94 (24)	
**Insulin Resistance**			0.635
Absent, n=487	120 (91)	367 (92)	
Present, n=43	12 (9)	31 (8)	
**Gestational Diabetes**			0.075
Absent, n=462	121 (92)	341 (86)	
Present, n=68	11 (8)	57 (14)	
**Hypothyroidism**			0.187
Absent, n=449	121 (92)	348 (87)	
Present, n=61	11 (8)	50 (13)	
**Pre-/Eclampsia, PIH (c)**			0.283
Absent, n=416	108 (82)	308 (77)	
Present, n=114	24 (18)	90 (23)	
**Anemia**			0.038
Absent, n=430	99 (75)	331 (83)	
Present, n=100	33 (25)	67 (17)	

(a) CF: Complementary Feeding; (b) IUGR: Intrauterine Growth Restriction; (c) PIH: Pregnancy-Induced Hypertension. *p*-value by chi square of Pearson.
